# Evaluating the Accuracy and Reliability of Large Language Models (ChatGPT, Claude, DeepSeek, Gemini, Grok, and Le Chat) in Answering Item-Analyzed Multiple-Choice Questions on Blood Physiology

**DOI:** 10.7759/cureus.81871

**Published:** 2025-04-08

**Authors:** Mayank Agarwal, Priyanka Sharma, Pinaki Wani

**Affiliations:** 1 Physiology, All India Institute of Medical Sciences, Raebareli, IND; 2 Physiology, School of Medical Sciences and Research, Greater Noida, IND

**Keywords:** artificial intelligence, blood physiology, chatgpt, claude, deepseek, grok, item analysis, large language models, medical education, multiple-choice questions

## Abstract

Background

Previous research has highlighted the potential of large language models (LLMs) in answering multiple-choice questions (MCQs) in medical physiology. However, their accuracy and reliability in specialized fields, such as blood physiology, remain underexplored. This study evaluates the performance of six free-to-use LLMs (ChatGPT, Claude, DeepSeek, Gemini, Grok, and Le Chat) in solving item-analyzed MCQs on blood physiology. The findings aim to assess their suitability as educational aids.

Methods

This cross-sectional study at the All India Institute of Medical Sciences, Raebareli, India, involved administering a 40-item MCQ test on blood physiology to 75 first-year medical students. Item analysis utilized the Difficulty Index (DIF I), Discrimination Index (DI), and Distractor Effectiveness (DE). Internal consistency was assessed with the Kuder-Richardson 20 (KR-20) coefficient. These 40 item-analyzed MCQs were presented to six selected LLMs (ChatGPT, Claude, DeepSeek, Gemini, Grok, Le Chat) available as standalone Android applications on March 19, 2025. Three independent users accessed each LLM simultaneously, uploading the compiled MCQs in a Portable Document Format (PDF) file. Accuracy was determined as the percentage of correct responses averaged across all three users. Reliability was measured as the percentage of MCQs consistently answered correctly by LLM to all three users. Descriptive statistics were presented as mean ± standard deviation and percentages. Pearson's correlation coefficient or Spearman's rho was used to evaluate the associations between variables, with p < 0.05 considered significant.

Results

Item analysis confirmed the validity and reliability of the assessment tool, with a DIF I of 63.2 ± 20.4, a DI of 0.38 ± 0.20, a DE of 66.7 ± 33.3, and a KR-20 of 0.804. Among LLMs, Claude 3.7 demonstrated the highest reliable accuracy (95%), followed by DeepSeek (93%), Grok 3 beta (93%), ChatGPT (90%), Gemini 2.0 (88%), and Mistral Le Chat (70%). No significant correlations were found between LLM performance and MCQ difficulty, discrimination power, or distractor effectiveness.

Conclusions

The MCQ assessment tool exhibited an appropriate difficulty level, strong discriminatory power, and adequately constructed distractors. LLMs, particularly Claude, DeepSeek, and Grok, demonstrated high accuracy and reliability in solving blood physiology MCQs, supporting their role as supplementary educational tools. LLMs handled questions of varying difficulty, discrimination power, and distractor effectiveness with similar competence. However, given occasional errors, they should be used alongside traditional teaching methods and expert supervision.

## Introduction

Large language models (LLMs) are rapidly transforming medical education. These generative artificial intelligence (AI) tools offer new ways to access and process information. Recent research has explored their potential as innovative teaching and learning aids [1,2]. However, their integration into medical education requires rigorous evaluation to ensure their accuracy and reliability in delivering domain-specific knowledge.

Medical physiology is the foundation of medical education, enabling students to understand the complex mechanisms regulating human health and disease [3]. Within this domain, blood physiology is a key module in the competency-based medical education (CBME) framework established by the National Medical Commission (NMC). This module covers essential concepts such as hemopoiesis, anemia, blood grouping, transfusion reactions, hemostasis, and immune responses [4]. An in-depth understanding of these concepts allows first-year medical students to connect physiological principles with clinical conditions in their future practice.

Multiple-choice questions (MCQs) remain a vital component of medical assessments due to their objectivity, scalability, and ability to evaluate higher-order thinking skills [5]. Item-analyzed MCQs ensure reliability and validity, ensuring accurate assessment while minimizing bias and ambiguity [6].

Recent studies suggest that LLMs, such as ChatGPT, can outperform medical undergraduate students in answering physiology MCQs [7-9]. While promising, these findings require further exploration in specialized modules such as blood physiology. LLMs can provide instant feedback, clarify complex topics, and generate practice questions [1-3,10-12]. However, their limitations may inadvertently misguide learners [1-3,10-12], particularly due to challenges in domain-specific accuracy [13,14]. Errors in their responses could lead to misconceptions, potentially limiting their effectiveness as educational tools.

While previous studies have evaluated LLMs' performance in medical physiology [3,8-12], their reliability in specialized areas such as blood physiology remains underexplored. The present study addresses this gap by evaluating six popular LLMs (ChatGPT, Claude, DeepSeek, Gemini, Grok, and Le Chat) in answering item-analyzed MCQs on blood physiology.

This study had two main objectives: (1) to perform an item analysis on blood physiology MCQs to ensure their quality and validity and (2) to compare the accuracy and reliability of multiple LLMs in answering these validated MCQs.

To the best of our knowledge, this is among the first studies to systematically compare the accuracy and reliability of multiple LLMs using item-analyzed, curriculum-aligned blood physiology questions. The findings of this study will guide educators in optimally integrating LLMs into medical education. This evaluation is particularly relevant for medical students and educators considering the use of LLMs as supplemental learning tools. By bridging technological advancements with practical educational applications, our research provides valuable insights into the evolving landscape of medical education.

## Materials and methods

Study setting, design, and ethical clearance

This cross-sectional study was conducted in the Department of Physiology at All India Institute of Medical Sciences (AIIMS), Raebareli, Uttar Pradesh, India. The institutional ethics committee granted ethical clearance for the research.

Selection criteria for LLMs

LLMs were selected based on their accessibility and practicality for student use. The inclusion criteria required models to be accessible without a paid subscription, available as standalone applications for Android smartphones, capable of processing Portable Document Format (PDF) files as input, and accessible via a Google account. Table 1 presents the six LLMs included in this study.

**Table 1:** LLMs involved in the study. LLMs: Large language models

LLM models	Developer	Android version
ChatGPT (Chat Generative Pre-trained Transformer)	OpenAI	1.2025.070
Claude 3.7 Sonnet	Anthropic	1.250310.7
DeepSeek	Liang Wenfeng (Hangzhou DeepSeek Artificial Intelligence Basic Technology Research Co., Ltd.)	1.1.3
Gemini 2.0 Flash	Google AI	1.0.686588308
Grok 3 beta	xAI	0.2.8
Le Chat	Mistral AI	1.0.10

Several models were excluded for specific reasons. Perplexity AI was excluded because it primarily functions as a search engine that integrates multiple LLMs, preventing an independent evaluation. Meta AI was omitted because it lacks a standalone application and does not support PDF file input when integrated into WhatsApp. Microsoft Copilot access required an exclusive Microsoft account; hence, it was excluded.

Item analysis of MCQs

The first author curated a set of 40 MCQs on blood physiology, incorporating both recall-based and higher-order cognitive questions. These validated MCQs were administered to 75 first-year Bachelor of Medicine and Bachelor of Surgery (MBBS) students at AIIMS Raebareli during an announced unit test in December 2023. Each MCQ had four options with a single correct answer. Students were allotted 40 minutes to complete the test. Each correct response was awarded one mark, with no negative marking for incorrect answers.

Students were ranked in descending order based on their scores. The top 27% (n = 20) were categorized as high achievers, and the bottom 27% (n = 20) were classified as low achievers [6]. Each MCQ was analyzed for Difficulty Index (DIF I), Discrimination Index (DI), and Distractor Effectiveness (DE). Table 2 outlines the criteria and interpretation for each parameter.

**Table 2:** Criteria and interpretation of item analysis parameters (DIF I, DI, and DE). H and L represent the number of students who answered the MCQ correctly in the high-achieving and low-achieving groups, respectively. N represents the total number of students in both groups, including those who did not respond. DIF I: Difficulty index; DI: Discrimination index; DE: Distractor effectiveness

Parameters of item-analysis	Use	Formula to calculate the score	Interpretation of the score		
DIF I	Assesses the difficulty level of an MCQ for a group of test-takers	((H + L) × 100) ÷ N	>70%: Very easy	30-70%: Acceptable to good	<30%: Very difficult
DI	Assesses the effectiveness of an MCQ in differentiating students with higher and lower cognitive abilities	(2 × (H – L)) ÷ N	≤ 0.20: Poor DI	0.21-0.34: Acceptable to good	≥0.35: Excellent
DE	Assesses the quality of incorrect options by evaluating the number of Non-Functional Distractors (NFDs) in an MCQ	NFDs are options chosen by fewer than 5% of total students	3 NFDs: Poor DE (0%)	2 to 1 NFD: Acceptable to good (33.3% for 2 NFDs and 66.6% for 1 NFD)	0 NFD: Excellent DE (100%)

The internal consistency reliability of the MCQ test was assessed using the Kuder-Richardson 20 (KR-20) coefficient, a measure specifically designed for dichotomous data and considered a special case of Cronbach's alpha. A KR-20 value higher than 0.8 suggests good reliability [6].

Evaluation of LLMs

The 40-item-analyzed MCQs were presented to the six selected LLMs on March 19, 2025. Three independent users accessed each LLM simultaneously using Android smartphones. All LLMs were evaluated under default settings in alphabetical order.

To ensure uniformity in testing conditions, all users uploaded a PDF containing 40 MCQs to each LLM, followed by a standardized prompt for response generation. This prompt included a contextual introduction, a general request, instructions on how the LLM should respond, and a specified output format [12]. The exact prompt was as follows: "Act as a medical college professor with expertise in Physiology. Use your knowledge and expertise to thoroughly analyze the provided multiple-choice questions (MCQs) and determine the correct answers. Provide the answers clearly and concisely by listing the MCQ number followed by the correct option (e.g., 1. a, 2. c). Explanations are not needed." We ensured that the Google account used to access each LLM was free of user-side pretraining data or chats.

The concepts of accuracy and reliability are fundamental to the integrity of measurement and data analysis across various disciplines. Accuracy refers to how close a measurement is to the true or accepted value. It indicates the extent to which a measurement aligns with a recognized standard or the actual value of the phenomenon being studied. Reliability, in contrast, refers to the consistency and repeatability of measurements under the same conditions. A reliable process produces similar results when repeated on the same object or subject under comparable circumstances. The key difference lies in their focus: accuracy concerns whether the measurement is correct, while reliability concerns whether the measurement remains consistent across repeated trials. In scientific contexts, accuracy relates to proximity to the true value, whereas reliability pertains to the consistency of results. In everyday use, the term "reliable" may imply both consistency and accuracy, which can lead to confusion. Therefore, understanding the context is essential. In this study, we have used the term reliability to encompass both accuracy and repeatability.

Accuracy was defined as the total number of correct responses provided by an LLM across three users. Each correct response was awarded one mark, while incorrect responses received zero. The percentage accuracy was calculated by dividing the average score of all three users by 40 and multiplying by 100. Reliability ensures that results are not due to random chance and can be reproduced under the same conditions. The reliability percentage was calculated by dividing the number of MCQs that were answered correctly for all three users by 40 and multiplying by 100.

Statistical analysis of data

Data were recorded using Microsoft Excel 365 (Microsoft Corporation, Redmond, WA). Statistical analyses were conducted using IBM SPSS Statistics for Windows (version 27.0; Released 2020; IBM Corp., Armonk, NY). Descriptive statistics were presented as mean ± standard deviation (SD), median ± inter-quartile range (IQR), percentages, and frequencies. Pearson's correlation coefficient was used for continuous data, while Spearman's rho was applied for categorical data to determine associations between variables, with p < 0.05 considered statistically significant.

## Results

MCQ item analysis

A total of 75 first-year MBBS students participated in the blood physiology MCQ test, which comprised 40 questions. The mean test score was 25.0 ± 6.0 (62.5 ± 14.9%), with scores ranging from 14 (35%) to 35 (87.5%). High achievers (n = 20) scored 32.7 ± 1.5 (81.8 ± 3.7%), while low achievers (n = 20) scored 17.6 ± 1.8 (44.0 ± 4.5%).

Item analysis of these MCQs revealed a mean DIF of 63.2 ± 20.4, indicating an overall moderate difficulty level. The mean DI was 0.38 ± 0.20, suggesting excellent differentiation between high- and low-achieving students. The median DE was 66.7 ± 33.3, reflecting adequately constructed distractors. Detailed results of the MCQ analysis are presented in Table 3.

**Table 3:** MCQ analysis for the DIF I, DI, and DE. MCQ: Multiple-choice question; NFD: Non-functional distractor; DIF I: Difficulty index; DI: Discrimination index; DE: Distractor effectiveness

MCQ (n=40) analysis indices	Description	Items (%)	Mean ± SD
DIF I	<30%	3 (7.5 %)	19.2 ± 5.2
	30-70%	23 (57.5%)	55.7 ± 7.9
	>70%	14 (35.0%)	85.0 ± 8.7
DI	≤0.20	9 (22.5%)	0.12 ± 0.08
	0.21-0.34	9 (22.5%)	0.27 ± 0.03
	≥0.35	22 (55.0%)	0.53 ± 0.12
DE	Total functional distractors	86 (71.7%)	72.5 ± 31.0
	Total NFDs	34 (28.3%)	
	Number of MCQs with 3 NFDs	3 (7.5%)	
	Number of MCQs with 2 NFDs	5 (12.5%)	
	Number of MCQs with 1 NFD	15 (37.5%)	
	Number of MCQs with 0 NFD	17 (42.5%)	

Among the 40 MCQs, 22 (55%) met all three quality criteria (DIF I: 30-70%, DI: > 0.2, and NFDs: ≤ 2) and were classified as acceptable to good. Of these, 11 (27.5%) achieved ideal status (DIF I: 30-70%, DI: ≥ 0.35, and NFD: 0) [6].

Correlation analysis revealed a weak, negative correlation between DIF I and DI (r = -0.219, p = 0.174), which was statistically insignificant. A weak, negative correlation was observed between DI and DE (r = -0.297, p = 0.063), nearing statistical significance. However, a strong, statistically significant negative correlation was obtained between DE and DIF I (r = -0.716, p < 0.001).

The KR-20 reliability coefficient for the test was 0.804. Removing any individual MCQ did not improve this reliability score.

Performance of LLMs

**Figure 1:**
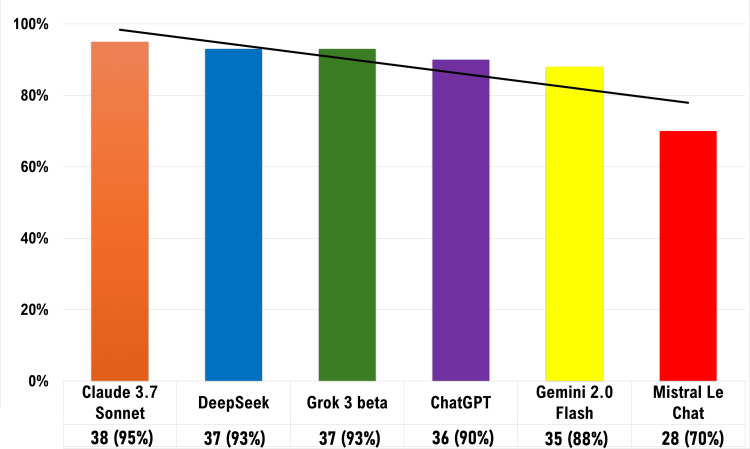
Reliability (with a trend line) of the various LLMs involved in the study. Reliability percentage indicates the percentage of MCQs correctly answered by LLMs to all three users. LLM: Large language model; MCQs: Multiple-choice questions

Table 4 presents the accuracy scores, and Figure 1 illustrates the reliability scores of the tested LLMs. Claude 3.7 Sonnet reliably answered 38 MCQs correctly, followed by DeepSeek (37), Grok 3 beta (37), ChatGPT (36), Gemini 2 Flash (35), and Mistral Le Chat (28). No significant association was found between LLM reliability and MCQ quality parameters (DIF I, DI, or DE).

**Table 4:** Accuracy of the various LLMs involved in the study. LLM: Large language model; MCQs: Multiple-choice questions

LLM model	Score for user 1	Score for user 2	Score for user 3	Average score (accuracy%)
Claude 3.7 Sonnet	38	38	38	38.00 (95%)
DeepSeek	37	37	38	37.33 (93%)
Grok 3 beta	37	37	38	37.33 (93%)
ChatGPT (Chat Generative Pre-trained Transformer)	36	37	36	36.33 (91%)
Gemini 2.0 Flash	37	37	35	36.33 (91%)
Le Chat	32	32	31	31.67 (79%)

All LLMs consistently answered MCQ number 7 incorrectly. Only Gemini answered MCQ number 27 correctly for all three users. Only Grok and Claude answered MCQ number 19 correctly for all three users. These three questions are displayed in Table 5. All MCQs, LLM responses, and item analysis data are included in the appendices to ensure data transparency.

**Table 5:** MCQs that LLMs frequently answer incorrectly. DIF I: Difficulty index; DI: Discrimination index; DE: Distractor effectiveness; LLMs: Large language models; MCQs: Multiple-choice questions; NFD: Non-functional distractor

MCQ number	Stem	Option ‘a’	Option ‘b’	Option ‘c’	Option ‘d’	Correct option	NFD	DE (%)	DIF I	DI
7	What is the most likely diagnosis for an adult male with an erythrocyte count of 2 million/mm^3^, haematocrit of 30%, and haemoglobin of 9.3 gm/dL?	Iron deficiency anaemia	Folic acid deficiency anaemia	Thalassemia	Sideroblastic anaemia	b	0	100	57.5	0.25
19	Both prothrombin time and activated partial thromboplastin time will NOT be increased in deficiency of which coagulation factor?	II	V	VIII	X	c	d	66.7	75	0.20
27	Erythropoietin plays the most vital role in the differentiation of:	Hematopoietic stem cells	Colony-forming units	Burst-forming units	Common myeloid progenitors	b	0	100	42.5	0.65

## Discussion

Our study evaluated the accuracy and reliability of six LLMs in solving item-analyzed MCQs on blood physiology. The findings indicate that Claude outperformed all other LLMs, with DeepSeek and Grok following closely. ChatGPT and Gemini demonstrated slightly lower reliability and accuracy, whereas Le Chat had the weakest performance. It is important to note that we did not apply any statistical tests to compare the performance of the LLMs, as all models, except Le Chat, performed at a similar level. Consequently, we relied on descriptive data to identify the best-performing model.

MCQ item analysis

The quality of the MCQ assessment tool was validated through item analysis, demonstrating good levels of DIF I, DI, DE, and reliability. In this study, 56% of the MCQs achieved an acceptable to good DIF I, which is lower than the 82% reported in a previous study [6]. Additionally, 78% of the MCQs exhibited an acceptable to excellent DI, closely aligning with the 80% reported earlier [6]. The proportion of non-functioning distractors (NFDs) was 72%, comparable to the 73% found in prior research [6]. More than half of the MCQs met all three quality criteria, and over a quarter reached the ideal standard, reflecting a slight improvement over the 20% previously reported [6]. Furthermore, the mean DIF I, DI, DE, and reliability observed in this study surpassed those documented in earlier research [6].

The significant negative correlation between DE and DIF I indicates that easier questions tended to have fewer effective distractors. Additionally, a weak positive correlation between DI and DE, approaching statistical significance, highlights the importance of well-structured distractors in differentiating among students with varying cognitive abilities. These findings align with established psychometric principles in test construction [6,15].

LLM performance

Our results align with recent studies demonstrating LLMs' strong performance in medical physiology examinations. Subramani et al. [7] found that ChatGPT scored 85% in 20 medical physiology MCQs, while Mondal et al. [8] reported that ChatGPT's performance surpassed the median student score. Similarly, Soulage et al. [9] demonstrated that ChatGPT outperformed medical students in a university physiology exam. However, our study extends beyond these findings in several important ways. First, we compared six different LLMs rather than focusing on a single model. Second, we used item-analyzed MCQs that met specific quality parameters. Third, we assessed both accuracy and reliability across multiple users.

Only Le Chat scored lower than the highest-achieving student. Claude's superior performance aligns with previous findings [12,16,17]. The performance of LLM did not show a significant correlation with the quality parameters of MCQs (DIF I, DI, DE). This suggests that LLMs can handle questions of varying difficulty, discrimination power, and distractor effectiveness with similar competence. This finding contrasts with a previous study that stated that LLM accuracy declines with increasing MCQ difficulty [18].

MCQ number seven presented a challenge for all tested LLMs. This question involved calculating the mean corpuscular volume (MCV = hematocrit ÷ RBC count in millions/mm³ × 10) and the mean corpuscular hemoglobin concentration (MCHC = hemoglobin in g/dL ÷ hematocrit × 100) for morphological classification of anemia. The correct answer was macrocytic normochromic anemia (MCV = 150 femtoliter, MCHC = 31%) caused by folic acid deficiency. All LLMs incorrectly classified it as iron deficiency anemia, consistent with prior research suggesting chatbots struggle with hypothetical scenarios [19].

MCQ 19 included a negative statement in the stem, which likely confused some LLMs. MCQ 27 is a factual question that appeared in the Indian medical postgraduate entrance examination a few years ago. The question presented two closely related options: the burst-forming unit (BFU) and the colony-forming unit (CFU). The correct answer is CFU, as it has more erythropoietin receptors and responds better to erythropoietin [20]. Students or instructors might have Googled the question; therefore, only Google Gemini can answer it accurately since it has access to Google's search database.

Wrong responses from generative AI can confuse medical students, leading them to learn incorrect medical facts or concepts. This is particularly concerning in a field where accuracy is critical, as it could result in poor clinical practice later on. To address this issue, it is important to validate AI tools thoroughly, teach students to critically evaluate AI outputs, and ensure human experts oversee AI-generated content.

Implications

The high performance of LLMs in blood physiology MCQs has a significant implication for medical education. These models could be valuable supplementary tools for students preparing for MCQ-based examinations. However, caution is warranted. Despite their high accuracy, even the best-performing model made errors in 5% of questions. In medical education, such errors could lead to misconceptions, potentially affecting students' understanding of critical concepts. Therefore, we recommend that students use LLMs as supplementary tools rather than primary learning resources. Based on the findings of the current study and previous research, we recommend that educators consider using these tools to generate practice questions, support student self-assessment, and scaffold explanations for challenging topics while ensuring that AI-generated content is reviewed for accuracy [3,7,8,10-12].

Future research should explore LLMs' ability to explain concepts rather than just providing correct answers to MCQs. This would help determine their potential as teaching tools beyond assessment aids. Additionally, advanced LLM capabilities, such as DeepSeek's DeepThink (R1), ChatGPT's Reason, Gemini's Deep Research and Flash Thinking, and Grok's DeepSearch and Think, are available for use without a paid subscription (though access may be limited) and should be explored further for their educational applications in medical training.

Limitations

Several limitations should be considered when interpreting our results. First, the study assessed LLMs using single best-answer MCQs. Performance might vary with other question formats, such as true or false questions, case-based scenarios, or open-ended responses. Second, we evaluated LLMs using a specific prompt. Different prompting techniques might yield different results. Future studies could explore various prompting strategies to optimize LLM performance. Third, our study focused exclusively on blood physiology. Performance might differ in other areas of medical physiology or clinical subjects. Future studies should investigate broader physiological and medical topics. Fourth, we conducted testing on a single day (March 19, 2025). LLMs undergo frequent updates, so their performance might change over time. Regular evaluations are necessary to track ongoing improvements or regressions. Finally, the study included only free-to-use LLMs available as Android applications. Premium or subscription-based models might offer different performance characteristics, possibly yielding higher accuracy and reliability.

## Conclusions

Our study demonstrates that modern LLMs, particularly Claude, DeepSeek, and Grok, show remarkable accuracy and reliability in answering item-analyzed blood physiology MCQs. These findings suggest that LLMs could be valuable supplementary medical education tools. However, even the best-performing models made errors, emphasizing the need for caution when using LLMs as learning aids. Students and educators should use these tools to complement, rather than replace, traditional teaching methods and expert guidance.

As LLM technology continues to evolve, its role in medical education must be continuously reassessed. Regular evaluation of LLM accuracy, reliability, and educational effectiveness will ensure that these powerful tools are optimally integrated into medical curricula while minimizing potential risks.

## Appendices

**Table 6:** Item-analyzed MCQs used in the study. DIF I: Difficulty index; DI: Discrimination index; DE: Distractor effectiveness; MCQ: Multiple-choice question; NFD: Non-functional distractor

MCQs	Correct Option	NFD	DE	DIF I	DI
1. Elevated eosinophil levels are typically observed in infections or infestations caused by: a. Aspergillus (fungus), b. Coronavirus, c. Tapeworm, d. Staphylococcus bacteria	c	0	100	62.5	0.35
2. The process of white blood cells crossing the endothelium is referred to as: a. Margination, b. Rolling, c. Adhesion, d. Diapedesis	d	a, c	33.3	92.5	0.15
3. Which enzyme induces the 'oxygen burst' to eliminate pathogens by neutrophils? a. Superoxide dismutase, b. Myeloperoxidase, c. NADPH oxidase, d. Catalase	c	0	100	45	0.5
4. Which of the following is the most likely outcome of chemotaxis induced by cells infected with bacteria? a. Unidirectional motion of leukocytes, b. Adhesion of erythrocytes to endothelium, c. Release of inflammatory mediators from basophil, d. Phagocytosis by lymphocytes	a	0	100	65	0.6
5. 'Major basic protein' is a significant component of granules within: a. Macrophage, b. Eosinophils, c. Basophil, d. Neutrophil	b	a	66.7	52.5	0.55
6. Acetone-free methanol in Leishman stain serves to: a. Fixes cells to slide, b. Stains the cellular components, c. Enhances metabolic and enzymatic activity, d. Washes the slide	a	0	100	25	0.1
7. What is the most likely diagnosis for an adult male with an erythrocyte count of 2 million/mm^3^, haematocrit of 30%, and haemoglobin of 9.3 gm/dL? a. Iron deficiency anaemia, b. Folic acid deficiency anaemia, c. Thalassemia, d. Sideroblastic anaemia	b	0	100	57.5	0.25
8. Bart haemoglobin is a tetramer of which polypeptide chain? a. Alpha, b. Beta, c. Gamma, d. Delta	c	0	100	52.5	0.35
9. Heterozygous sickle cell anaemia is expected to offer protection against: a. Glucose-6-phosphate dehydrogenase (G6PD) deficiency, b. Plasmodium parasite, c. Thalassemia, d. Dengue fever	b	a, c, d	0	100	0
10. Which of the following findings are correct for iron deficiency anaemia: a. Elevated total iron binding capacity, serum ferritin, and transferrin saturation, b. Reduced total iron binding capacity, serum ferritin, and transferrin saturation, c. Elevated total iron binding capacity and transferrin saturation, but reduced serum ferritin, d. Elevated total iron binding capacity, but reduced serum ferritin and transferrin saturation	d	a	66.7	47.5	0.45
11. Causes of megaloblastic anaemia include all of the following EXCEPT: a. Defects in DNA synthesis, b. Folic acid deficiency, c. Lead toxicity, d. Vitamin B12 deficiency	c	b, d	33.3	87.5	0.25
12. Find the FALSE statement regarding megaloblastic anaemia: a. Hyper-segmented neutrophils are the earliest manifestation, b. Reticulocyte count decreased, c. Mean corpuscular volume is increased, d. Mean corpuscular haemoglobin concentration is increased	d	c	66.7	60	0.6
13. Iron absorption is increased by: a. Phytate, b. Tannate, c. Alkali, d. Ascorbic acid	d	a, b	33.3	87.5	0.25
14. True about Haemophilia B is: a. Autosomal recessive, b. Fresh frozen plasma for treatment, c. X-linked dominant, d. Prothrombin time is raised	b	0	100	55	0.5
15. Which of the following occurs earliest in response to vascular trauma? a. Constriction of the traumatised vessel, b. Adhesion of platelets to damaged endothelium, c. Aggregation of platelets, d. Dilatation of the traumatised vessel	a	c	66.7	80	0.4
16. What causes platelets to adhere to the injured endothelium of the blood vessel wall? a. Coagulation factor VIII, b. Fibrinogen, c. von Willebrand factor, d. Coagulation factor III	c	a, b, d	0	90	0.2
17. Platelet-derived growth factor (PDGF) is present in which granules of platelets? a. Alpha, b. Lysosomes, c. Delta (Dense), d. Peroxisomes	a	b	66.7	62.5	0.75
18. Correct regarding von Willebrand disease is: a. Activated partial thromboplastin time remains unaffected, b. Bleeding time remains unaffected, c. Prothrombin time remains unaffected, d. All of the above statements are true	c	0	100	17.5	0.15
19. Both prothrombin time and activated partial thromboplastin time will NOT be increased in deficiency of which coagulation factor? a. II, b. V, c. VIII, d. X	c	d	66.7	75	0.2
20. Which of the following is utilised to monitor warfarin therapy: a. PT (prothrombin time), b. BT (bleeding time), c. PTT (partial thromboplastin time), d. TT (thrombin time)	a	0	100	52.5	0.45
21. Which of the following characteristics is associated with coagulation factor XIII deficiency? a. Clot dissolves quickly, b. Clot remains stable, c. Clot formation does not occur, d. Clotting time is prolonged	a	0	100	62.5	0.45
22. All of the statements provided regarding blood coagulation are correct, EXCEPT: a. Factor X is involved in both the intrinsic and extrinsic pathways, b. Activation of the extrinsic pathway occurs upon contact with negatively charged surfaces, c. Intrinsic pathway can be activated outside the human body, d. Calcium is essential for several steps of coagulation	b	d	66.7	67.5	0.65
23. Which pair of regulatory proteins form a complex responsible for breaking down activated coagulation factors V and VIII? a. Tissue factor pathway inhibitor and tissue plasminogen activator, b. Antithrombin III and heparin, c. Thrombomodulin and plasmin, d. Activated protein C and protein S	d	a	66.7	62.5	0.45
24. Aspirin primarily decreases the chance of blood clot formation within blood vessels by: a. Impeding the extrinsic clotting pathway, b. Impeding the intrinsic clotting pathway, c. Impeding the platelet function, d. Stimulating anticoagulant synthesis	c	0	100	45	0.6
25. Which transfusion is most likely to cause the most severe transfusion reaction in a patient who has never undergone a transfusion before? a. Transfusing A+ packed cells to an O- patient, b. Transfusing A+ packed cells to an A- patient, c. Transfusing A- packed cells to an A+ patient, d. Transfusing A- packed cells to an AB+ patient	a	d	66.7	82.5	0.25
26. Heparin primarily prevents blood clotting by: a. Activating Antithrombin III, b. Binding to and inhibiting tissue factor, c. Binding to free calcium, d. Inhibiting platelet-activating factor	a	0	100	65	0.7
27. Erythropoietin plays the most vital role in the differentiation of: a. Hematopoietic stem cells, b. Colony-forming units, c. Burst-forming units, d. Common myeloid progenitors	b	0	100	42.5	0.65
28. The type of jaundice associated with elevated urobilinogen but absence of bilirubin in urine is: a. Acholuric (pre-hepatic), b. Obstructive (post-hepatic), c. Hepatitis associated (hepatic), d. Both B and C	a	d	66.7	55	0.7
29. For a patient prescribed the oral anticoagulant warfarin, an international normalised ratio (INR) of 1.1 suggests that the level of anticoagulation is: a. Sufficient and requires no alteration to the warfarin dose, b. Insufficient and warrants an increase in the warfarin dose, c. Insufficient and necessitates a reduction in the warfarin dose, d. Excessive and necessitates a vitamin K injection	b	0	100	45	0.4
30. Which of the following types of haemoglobin is generally ABSENT in a healthy adult? a. Haemoglobin having two alpha and two beta protein subunits, b. Haemoglobin having two alpha and two gamma protein subunits, c. Haemoglobin having two alpha and two delta protein subunits, d. Haemoglobin having two alpha and two epsilon protein subunits	d	a	66.7	62.5	0.55
31. A patient with a history of a chronic inflammatory condition presents with elevated levels of acute-phase reactants in the blood. Which of the following plasma proteins is likely to be increased in response to inflammation? a. Albumin, b. Fibrinogen, c. Haemoglobin, d. Globulin	b	c	66.7	15	0.2
32. A 40-year-old male shows generalised oedema. Laboratory tests indicate a reversal of the albumin: globulin ratio. What is the most probable cause of oedema? a. Impaired liver synthesis of albumin, b. Increased capillary permeability, c. Altered kidney function, d. Lymphatic obstruction	a	d	66.7	57.5	0.05
33. A 40-year-old female is diagnosed with polycythaemia vera. What primary abnormality in erythropoiesis characterises this condition? a. Impaired synthesis of erythropoietin, b. Uncontrolled proliferation of erythroid precursors, c. High levels of erythropoietin, d. Increased destruction of mature red blood cells	b	0	100	77.5	0.25
34. A patient diagnosed with chronic lymphocytic leukaemia displays symptoms of anaemia. What pathophysiological mechanism is primarily responsible for the anaemia in this patient? a. Invasion of leukemic cells into erythroid precursor cells, b. Increased production of erythropoietin, c. Impaired absorption of iron, d. Accelerated destruction of red blood cells	a	c	66.7	75	0.5
35. A 45-year-old female is found to have increased levels of hepcidin during chronic inflammation. What effect does hepcidin have on iron availability for erythropoiesis? a. Increases iron absorption, b. Decreases iron absorption, c. Facilitates iron release from macrophages, d. Promotes iron sequestration in hepatocytes	b	0	100	42.5	0.25
36. A 38-year-old woman exhibits haemolysis, causing the release of free haemoglobin into the bloodstream. What scavenger protein binds to free haemoglobin, preventing renal excretion, and facilitating its degradation? a. Haptoglobin, b. Hemopexin, c. Ferritin, d. Biliverdin	a	b, c	33.3	85	0.3
37. An adult woman suffering from heavy menstrual bleeding had a normal platelet count. However, her platelets exhibit an impaired response to the von Willebrand factor. What platelet function defect is likely causing her tendency to bleed excessively? a. Decreased platelet adhesion, b. Impaired platelet aggregation, c. Altered platelet secretion, d. Enhanced platelet production	a	c, d	33.3	85	0.3
38. A woman with blood type O is the mother of two children with blood types A and B, respectively. Assuming a monogamous relationship, determine the possible blood type of the father. a. A, b. B, c. AB, d. O	c	a, b, d	0	100	0
39. A patient is prescribed a medication that enhances the conversion of plasminogen to plasmin. What therapeutic effect does this medication have on fibrinolysis? a. Inhibition of fibrinolysis, b. Stimulation of fibrinolysis, c. Prevention of fibrin clot formation, d. Acceleration of fibrin clot stabilisation	b	c	66.7	72.5	0.55
40. A patient with a past trauma history was hospitalised due to fibrinolytic bleeding. Laboratory assessments show increased levels of fibrin degradation products. Deficiency of which of the following is most likely contributing to this bleeding disorder? a. Plasminogen activator inhibitor-1, b. Activated protein C, c. Tissue plasminogen activator, d. Plasmin	a	0	100	60	0.3

**Table 7:** LLM responses to three users. Correct response has been labelled as '1'. LLM: Large language model; MCQ: Multiple-choice question

MCQ number	DeepSeek v1.1.3			Gemini 2.0 v1.0.686588308			Claude 3.7 v1.250310.7			ChatGPT v1.2025.070			Mistral Le Chat v1.0.10			Grok 3 beta v0.2.8			Key
	User 1	User 2	User 3	User 1	User 2	User 3	User 1	User 2	User 3	User 1	User 2	User 3	User 1	User 2	User 3	User 1	User 2	User 3	
1	1	1	1	1	1	1	1	1	1	1	1	1	a	a	1	1	1	1	c
2	1	1	1	1	1	1	1	1	1	1	1	1	1	1	1	1	1	1	d
3	1	1	1	1	1	1	1	1	1	1	1	1	1	1	1	1	1	1	c
4	1	1	1	1	1	1	1	1	1	1	1	1	d	d	d	1	1	1	a
5	1	1	1	1	1	1	1	1	1	1	1	1	1	1	1	1	1	1	b
6	1	1	1	1	1	1	1	1	1	1	1	1	1	1	1	1	1	1	a
7	a	a	a	a	a	a	a	a	a	a	a	a	a	a	a	a	a	a	b
8	1	1	1	d	1	d	1	1	1	1	1	1	1	1	1	1	1	1	c
9	1	1	1	1	1	1	1	1	1	1	1	1	1	1	1	1	1	1	b
10	1	1	1	1	1	1	1	1	1	1	1	1	1	1	1	1	1	1	d
11	1	1	1	1	1	1	1	1	1	1	1	1	1	1	1	1	1	1	c
12	1	1	1	1	1	1	1	1	1	1	1	1	a	1	1	1	1	1	d
13	1	1	1	1	1	1	1	1	1	1	1	1	1	1	1	1	1	1	d
14	1	1	1	1	1	1	1	1	1	1	1	1	1	1	d	1	1	1	b
15	1	1	1	1	1	1	1	1	1	1	1	1	1	1	1	1	1	1	a
16	1	1	1	1	1	1	1	1	1	1	1	1	1	1	1	1	1	1	c
17	1	1	1	1	1	1	1	1	1	1	1	1	1	1	1	1	1	1	a
18	1	1	1	1	1	1	1	1	1	1	1	1	d	d	d	1	1	1	c
19	a	a	1	1	d	none	1	1	1	a	a	a	b	b	b	1	1	1	c
20	1	1	1	1	1	1	1	1	1	1	1	1	1	1	1	1	1	1	a
21	1	1	1	1	1	1	1	1	1	1	1	1	1	1	1	1	1	1	a
22	1	1	1	1	1	1	1	1	1	1	1	1	1	1	1	1	1	1	b
23	1	1	1	1	1	1	1	1	1	1	1	1	1	1	1	1	1	1	d
24	1	1	1	1	1	1	1	1	1	1	1	1	1	1	1	1	1	1	c
25	1	1	1	1	1	1	1	1	1	1	1	1	1	1	1	1	1	1	a
26	1	1	1	1	1	1	1	1	1	1	1	1	1	1	1	1	1	1	a
27	c	c	c	1	1	1	c	c	c	c	c	a	1	1	d	c	c	c	b
28	1	1	1	1	1	1	1	1	1	1	1	1	1	1	1	1	1	1	a
29	1	1	1	1	1	a	1	1	1	1	1	1	1	1	1	1	1	1	b
30	1	1	1	1	1	1	1	1	1	1	1	1	1	1	b	b	b	1	d
31	1	1	1	1	1	1	1	1	1	1	1	1	1	1	1	1	1	1	b
32	1	1	1	1	1	1	1	1	1	1	1	1	1	1	1	1	1	1	a
33	1	1	1	1	1	1	1	1	1	1	1	1	1	1	1	1	1	1	b
34	1	1	1	d	d	d	1	1	1	1	1	1	d	d	d	1	1	1	a
35	1	1	1	1	1	1	1	1	1	d	1	d	d	d	d	1	1	1	b
36	1	1	1	1	1	1	1	1	1	1	1	1	1	1	1	1	1	1	a
37	1	1	1	1	1	1	1	1	1	1	1	1	1	b	1	1	1	1	a
38	1	1	1	1	1	1	1	1	1	1	1	1	1	1	1	1	1	1	c
39	1	1	1	1	1	1	1	1	1	1	1	1	1	1	1	1	1	1	b
40	1	1	1	1	1	1	1	1	1	1	1	1	1	1	1	1	1	1	a
